# Age 23 years + oral health questionnaire in Avon Longitudinal Study of Parents and Children.

**DOI:** 10.12688/wellcomeopenres.14159.2

**Published:** 2018-04-30

**Authors:** Tom Dudding, Simon Haworth, Jonathan Sandy, Nicholas J. Timpson

**Affiliations:** 1 MRC Integrative Epidemiology Unit, University of Bristol, Bristol, BS8 2BN, UK; 2 Bristol Dental School, University of Bristol, Bristol, BS1 2LY, UK; 3 Faculty of Health Sciences, University of Bristol, Bristol, BS2 8DZ, UK

**Keywords:** ALSPAC, Oral Health, Dental, Dental Caries, Pericoronitis, Oral Ulcers, Wisdom Teeth

## Abstract

Oral health data in large longitudinal cohort studies is rarely collected at multiple time-points. This type of data is important for assessing oral health trajectories and their determinants. This data resource includes self-report questionnaire data on up to 4,222 young adults at approximately 23 years of age from the Avon Longitudinal Study of Parents and Children (ALSPAC). The resource includes questions on dental attendance, tooth restorations and extractions, third molars (wisdom teeth) and mouth ulcers. This round of data collection follows on from similar questionnaires at ages 7, 10 and 17 years. The ALSPAC study provides an opportunity to combine this oral health data with extensive phenotype, genetic, epigenetic and metabolomic data from the participants, their mothers and fathers.

## Introduction

The Avon Longitudinal Study of Parents and Children (ALSPAC) is a longitudinal birth cohort that recruited pregnant women living near Bristol, UK with an estimated delivery date between 1991 and 1992 ^1^. The study includes extensive phenotypic, genetic, epigenetic and metabolomic data on the mothers, fathers and children and follow up is ongoing. Information on the oral health of the children (now young adults) has been collected throughout the study by means of focussed questionnaires at age 7, 10 and 17 years. A smaller subset of participants also received clinical examinations at three time points before the age of 7 years. This dataset relates to the “Teeth” section of the “Me @ 23+” questionnaire that gathered information pertaining to the oral health of participants with the aim of allowing continued longitudinal assessment of their oral health. The questionnaire was designed to address or contribute to 4 research questions:

1) Can oral health in adolescence predict poor health outcomes in later life and if so at what stage is this detectable?

2) What are the major genetic and environmental risk factors for pericoronitis (infection of the gum area around wisdom teeth)?

3) At what stage do oral hygiene behaviours and beliefs predict periodontal outcomes? Are patterns of behaviour in childhood or early adult life more important?

4) What are the major genetic and environmental risk factors for mouth ulcers?

## Methods

ALSPAC recruited 14,541 pregnant women resident in Avon, UK (former county covering Bristol and the surrounding areas in the South West UK) with expected dates of delivery 1st April 1991 to 31st December 1992. 14,541 is the initial number of pregnancies for which the mother enrolled in the ALSPAC study and had either returned at least one questionnaire or attended a “Children in Focus” clinic by 19/07/99. Of these initial pregnancies, there were a total of 14,676 foetuses, resulting in 14,062 live births and 13,988 children who were alive at 1 year of age. When the oldest children were approximately 7 years of age, an attempt was made to bolster the initial sample with eligible cases who had failed to join the study originally. As a result, when considering variables collected from the age of seven onwards (and potentially abstracted from obstetric notes) there are data available for more than the 14,541 pregnancies mentioned above.

The number of new pregnancies not in the initial sample (known as Phase I enrolment) that are currently represented on the built files and reflecting enrolment status at the age of 18 is 706 (452 and 254 recruited during Phases II and III respectively), resulting in an additional 713 children being enrolled. The phases of enrolment are described in more detail in the cohort profile paper ^1, 2^.

The total sample size for analyses using any data collected after the age of seven is therefore 15,247 pregnancies, resulting in 15,458 foetuses. Of this total sample of 15,458 foetuses, 14,775 were live births and 14,701 were alive at 1 year of age.

The data included in this resource were generated from a questionnaire that the ALSPAC children (now young adults) completed at approximately 23 years of age (“Me @ 23+”). The questionnaire included 13 sections (A to M). Oral health questions were in section J (“Teeth”).

Where oral health topics had been asked before in previous ALSPAC questionnaires the same questions were used for consistency. Questions about third molars (wisdom teeth) attempted to identify whether participants had their wisdom teeth present and whether they had caused problems and were adapted from the UK National Third Molar Audit ^3^.

The questionnaire was available to complete in online or paper format between November 2015 and September 2016. Completed paper questionnaires were read using Cardiff TeleForm version 10.1 (Autonomy Corporation plc, Cambridge, England), data collection for the online questionnaires was collected and managed using REDCap electronic data capture tools ^4^ hosted at the University of Bristol.

Please note that the study website contains details of all the data that is available through a fully searchable data dictionary.

A version of the relevant section of the questionnaire is provided in Supplementary File 1.

**Figure 1.**
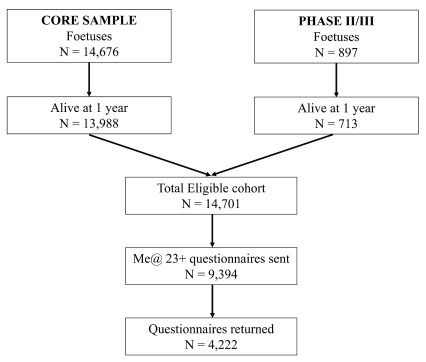
Recruitment of participants to the Avon Longitudinal Study of Parents and Children (ALSPAC).

There are a total of 15,573 records on this built file with 4,222 having returned a completed questionnaire. This number is made up of the 14,676 foetuses in the core ALSPAC sample plus 897 eligible children not in the core sample (regardless of whether or not the Me @ 23+ questionnaire was sent out to them or whether they were returned). This questionnaire was completed by 309 of these 897 children not in the core sample. Of the 14,676 foetuses in the core ALSPAC sample, 14,062 were live born. The Me @ 23+ questionnaire was sent out to 9,394 live born children within the ALSPAC cohort (60.3% of total sample). As of 30th of September 2016, 4,222 completed questionnaires had been returned (45% of those sent) ( Figure 1).

Note that 3 of the 4,222 completed questionnaires belong to children from triplet or quadruplet pregnancies, all of whom are in the core sample. For reasons of confidentiality the data from these questionnaires are not available. All variables have been set to missing for these triplets and quadruplets.

The questionnaire has been split into four datasets. Each dataset is described below, the questions are stated and the data summarised in tables alongside the matching ALSPAC variable code (e.g. YPC1950).

### Dataset 1. Visiting your dentist and tooth decay

This section asked participants about visiting the dentist, orthodontics and previous fillings and extractions. Where participants were asked to assess the current state of their own teeth they were encouraged to use a mirror, a diagrammatic representation of the mouth was included in the questionnaire to assist with accurate identification of teeth ( Supplementary File 1).

**YPC1950.** Length of time since respondent last went to the dentist ( Table 1).

**Table 1.** YPC1950. Length of time since respondent last went to the dentist.

Variable name: YPC1950	Frequency	Percent	Cumulative Percent
0 Never been	5	0.1	0.1
1 In the past year	2571	62.8	62.9
2 Between 1 and 2 years ago	759	18.5	81.5
3 More than 2 years ago	713	17.4	98.9
9 Don't know	46	1.1	100.0
Total	4094	100.0	

**YPC1960.** Reason respondent usually goes to the dentist ( Table 2).

**Table 2.** YPC1960. Reason respondent usually goes to the dentist.

Variable name: YPC1960	Frequency	Percent	Cumulative Percent
0 Never goes to the dentist	112	2.7	2.7
1 Regular routine check- ups (up to every 2 years)	2670	65.2	68.0
2 Occasional check-up (less than every 2 years)	686	16.8	84.8
3 Only when has trouble with teeth	554	13.5	98.3
4 Another reason	36	0.9	99.2
9 Don't know	34	0.8	100.0
Total	4092	100.0	

**YPC1970.** Any of respondent’s teeth taken out for braces/traintracks/orthodontics ( Table 3).

**Table 3.** YPC1970. Any of respondent's teeth taken out for braces/traintracks/orthodontics.

Variable name: YPC1970	Frequency	Percent	Cumulative Percent
0 No	2891	71.5	71.5
1 Yes	1152	28.5	100.0
Total	4043	100.0	

**YPC1980-YPC2007.** Respondent's teeth that have fillings (28 variables).

Each of the 28 variables are associated with a single tooth. Each variable is coded 1 if the participant reported having a filling or other restoration in that tooth. Table 4 summarises the numbers and percentage with fillings/restorations for each tooth. These tables present right and left alongside each other for ease of comparison.

**Table 4.** YPC1980-YPC2031. Reported frequency (%) of fillings and dental extractions for each tooth.

RIGHT					LEFT				
TOOTH (FDI)	FILLING		EXTRACTION		TOOTH	FILLING		EXTRACTION	
	N	%	N	%		N	%	N	%
UR1 (11)	159	3.9	21	0.5	UL1 (21)	174	4.2	18	0.4
UR2 (12)	83	2.0	62	1.5	UL2 (22)	92	2.2	66	1.6
UR3 (13)	70	1.7	66	1.6	UL3 (23)	62	1.5	71	1.7
UR4 (14)	110	2.7	153	3.7	UL4 (24)	99	2.4	169	4.1
UR5 (15)	251	6.1	139	3.4	UL5 (25)	237	5.8	145	3.5
UR6 (16)	614	15.0	102	2.5	UL6 (26)	637	15.5	101	2.5
UR7 (17)	413	10.1	126	3.1	UL7 (27)	471	11.5	127	3.1
LR1 (41)	42	1.0	9	0.2	LL1 (31)	61	1.5	22	0.5
LR2 (42)	52	1.3	21	0.5	LL2 (32)	63	1.5	16	0.4
LR3 (43)	52	1.3	42	1.0	LL3 (33)	62	1.5	43	1.1
LR4 (44)	72	1.8	92	2.2	LL4 (34)	84	2.1	90	2.2
LR5 (45)	175	4.3	131	3.2	LL5 (35)	203	5.0	140	3.4
LR6 (46)	706	17.2	92	2.2	LL6 (36)	724	17.7	102	2.5
LR7 (47)	576	14.0	134	3.3	LL7 (37)	562	13.7	134	3.3

U*# = Upper, L*# = Lower, *R# = Right, *L# = Left, number(#) indicates the tooth number from the midline, e.g. UR3 = Upper Right 3 – third tooth from midline on the upper right. FDI = Federation Dentaire Internationale numbering system.

**YPC2010-YPC2031.** Respondent's teeth that have been taken out (28 variables).

Each of the 28 variables are associated with a single tooth. Each variable is coded 1 if the participant reported having had their tooth taken out (extracted). Is should be noted that participants may have marked unerupted or congenitally absent teeth as ‘taken out’. Table 4 summarises the numbers and percentage with a tooth extracted for each tooth. These tables present right and left alongside each other for ease of comparison.

### Dataset 2: Third molars (wisdom teeth)

This section asked participants about their third molars (wisdom teeth). Participants were asked to indicate if each of their wisdom teeth has come through (erupted) and if it had, whether or not it had caused problems. Further questions asked more details about the nature and frequency of wisdom tooth problems and about treatments for wisdom tooth problems.

**YPC2040-YPC2043:** Respondent’s wisdom teeth that haven’t come through (4 variables).

Participants were asked to indicate if each of their 4 wisdom teeth (third molars) had or had not come through (erupted). Each variable is coded 1 if the wisdom had **not** come through. Table 5 summarises the responses for each wisdom tooth. NB: Percentages are based on total number of participants who answered the questionnaire, not all participants answered this question for each tooth and this is indicated by the missing column.

**Table 5.** YPC2040-YPC2063. Frequency (%) of respondent’s wisdom teeth that were unerupted, erupted with problems or erupted without problems.

TOOTH (FDI)	UNERUPTED		ERUPTED, PROBLEMS		ERUPTED, NO PROBLEMS		MISSING DATA	
	N	%	N	%	N	%	N	%
UR8 (18)	1333	32.5	1351	32.9	476	11.6	1062	25.2
UL8 (28)	1355	33.0	1327	32.3	494	12.0	1046	24.8
LL8 (38)	1326	32.3	1175	28.6	640	15.6	1081	25.6
LR8 (48)	1350	32.9	1162	28.3	669	16.3	1041	24.7

U*=Upper, L*=Lower, *R=Right, *L=Left, e.g. UR = Upper Right. FDI = Federation Dentaire Internationale numbering system.

**YPC2050-YPC2053:** Respondent's wisdom teeth that have come through and not caused problems (4 variables).

Participants were asked to indicate if each of their 4 wisdom teeth (third molars) had come through and **had not** caused problems. Each variable is coded 1 if the wisdom had **not** come through. Table 5 summarises the responses for each wisdom tooth. NB: Percentages are based on total number of participants who answered the questionnaire, not all participants answered this question for each tooth and this is indicated by the missing column.

**YPC2060-YPC2063:** Respondent's wisdom teeth that have come through and caused problems or pain, even if these teeth have now been removed (4 variables).

Participants were asked to indicate if each of their 4 wisdom teeth (third molars) had come through and **had** caused problems or pain, even if these teeth had now been removed. Each variable is coded 1 if the wisdom had **not** come through. Table 5 summarises the responses for each wisdom tooth. NB: Percentages are based on total number of participants who answered the questionnaire, not all participants answered this question for each tooth and this is indicated by the missing column.

**YPC2070:** Number of times respondent has had pain from their wisdom teeth ( Table 6).

**Table 6.** YPC2070. Number of times respondent has had pain from their wisdom teeth.

Variable name: YPC2070	Frequency	Percent	Cumulative Percent
0 Never	1522	41.4	41.4
1 1	345	9.4	50.8
2 2-3	674	18.3	69.1
3 3-4	352	9.6	78.7
4 5 or more times	612	16.6	95.3
9 Don't know	173	4.7	100.0
Total	3678	100.0	

**YPC2071:** Number of times respondent has had a course of antibiotics for problems with their wisdom teeth ( Table 7).

**Table 7.** YPC2071. Number of times respondent has had a course of antibiotics for problems with their wisdom teeth.

Variable name: YPC2071	Frequency	Percent	Cumulative Percent
0 Never	3133	85.6	85.6
1 1	286	7.8	93.4
2 2-3	115	3.1	96.6
3 3-4	35	1.0	97.5
4 5 or more times	40	1.1	98.6
9 Don't know	50	1.4	100.0
Total	3659	100.0	

**YPC2072:** Number of times respondent has had facial swelling from their wisdom teeth ( Table 8).

**Table 8.** YPC2072. Number of times respondent has had facial swelling from their wisdom teeth.

Variable name: YPC2072	Frequency	Percent	Cumulative Percent
0 Never	3013	82.4	82.4
1 1	333	9.1	91.5
2 2-3	154	4.2	95.8
3 3-4	49	1.3	97.1
4 5 or more times	50	1.4	98.5
9 Don't know	56	1.5	100.0
Total	3655	100.0	

**YPC2080:** Respondent has ever had to stay in a hospital bed, either during the day or overnight, because of problems from their wisdom teeth ( Table 9).

**Table 9.** YPC2080. Respondent has ever had to stay in a hospital bed, either during the day or overnight, because of problems from their wisdom teeth.

Variable name: YPC2080	Frequency	Percent	Cumulative Percent
0 No	3562	95.9	95.9
1 Yes	126	3.4	99.3
9 Don't know	26	0.7	100.0
Total	3714	100.0	

**YPC2090:** Respondent has had any wisdom teeth removed ( Table 10).

**Table 10.** YPC2090. Respondent has had any wisdom teeth removed.

Variable name: YPC2090	Frequency	Percent	Cumulative Percent
0 No	3288	88.5	88.5
1 Yes	368	9.9	98.4
9 Don't know	58	1.6	100.0
Total	3714	100.0	

**YPC2100:** Respondent has had any other treatment to their wisdom teeth when they were causing pain, like cleaning around the gum or removing part of the gum ( Table 11).

**Table 11.** YPC2100. Respondent has had any other treatment to their wisdom teeth when they were causing pain, like cleaning around the gum or removing part of the gum.

Variable name: YPC2100	Frequency	Percent	Cumulative Percent
0 No	3376	91.6	91.6
1 Yes	253	6.9	98.5
9 Don't know	56	1.5	100.0
Total	3685	100.0	

### Dataset 3: Mouth ulcers

This section asked participants about whether they had ever had mouth ulcers, the age at onset and the frequency of their occurrence.

**YPC2110:** Respondent ever had mouth ulcers ( Table 12).

**Table 12.** YPC2110. Respondent ever had mouth ulcers.

Variable name: YPC2110	Frequency	Percent	Cumulative Percent
0 No	1079	26.6	26.6
1 Yes, but only once or twice	1119	27.6	54.2
2 Yes, on several occasions	1751	43.2	97.4
9 Don't know	104	2.6	100.0
Total	4053	100.0	

**YPC2111:** Age when respondent first noticed that they had mouth ulcers ( Table 13).

**Table 13.** YPC2111. Age when respondent first noticed that they had mouth ulcers.

Variable name: YPC2111	Frequency	Percent	Cumulative Percent
1 Before was a teenager (or under 12 years)	922	31.4	31.4
2 While a teenager (13-19)	1251	42.7	74.1
3 In 20s	250	8.5	82.6
9 Don't remember	509	17.4	100.0
Total	2932	100.0	

**YPC2112:** Frequency respondent gets mouth ulcers ( Table 14).

**Table 14.** YPC2112. Frequency respondent gets mouth ulcers.

Variable name: YPC2112	Frequency	Percent	Cumulative Percent
1 Every month	222	7.5	7.5
2 Every 2-3 months	496	16.9	24.4
3 At least once every 6 months	612	20.8	45.2
4 At least once a year	496	16.9	62.1
5 Less than yearly	879	29.9	92.0
9 Don't remember	236	8.0	100.0
Total	2941	100.0	

**Table 15.** Valid and missing responses.

Variable name:	Variable topic	Valid (%)	Missing (%)
YPC1950	Length of time since respondent last went to the dentist	4094 (97.0)	128 (3.0)
YPC1960	Reason respondent usually goes to the dentist	4092 (96.9)	130 (3.1)
YPC1970	Any of respondent’s teeth taken out for braces/traintracks/orthodontics	4043 (95.8)	179 (4.2)
YPC2070	Number of times respondent has had pain from their wisdom teeth	3678 (87.1)	544 (12.9)
YPC2071	Number of times respondent has had a course of antibiotics for problems with their wisdom teeth	3659 (86.7)	563 (13.3)
YPC2072	Number of times respondent has had facial swelling from their wisdom teeth	3655 (86.6)	567 (13.4)
YPC2080	Respondent has ever had to stay in a hospital bed, either during the day or overnight, because of problems from their wisdom teeth	3714 (88.0)	508 (12.0)
YPC2090	Respondent has had any wisdom teeth removed	3714 (88.0)	508 (12.0)
YPC2100	Respondent has had any other treatment to their wisdom teeth when they were causing pain, like cleaning around the gum or removing part of the gum	3685 (87.3)	537 (12.7)
YPC2110	Respondent ever had mouth ulcers	4053 (96.0)	169 (4.0)
YPC2111	Age when respondent first noticed that they had mouth ulcers *(Count excludes those* *that answered “Don’t remember to YPC2111, N = 509; Percentage relates to those that* *answered “Yes,…” to YPC110, N = 2,870)*	2423 (84.4)	447 (15.6)
YPC2112	Frequency respondent gets mouth *ulcers (Count excludes those that answered “Don’t* *remember to YPC2112, N = 236; Percentage relates to those that answered “Yes,…” to* *YPC110, N = 2,870)*	2705 (94.3)	165 (5.7)

### Dataset validation

The number of valid and missing responses for variables, except those requiring the use of the mouth diagram and mirror (YPC1980-YPC2031 and YPC2040-YPC2043), is shown in Table 15.

During data cleaning it became apparent that some participants (less than 2.2%) had logical contradictions relating to some of the questions about wisdom teeth problems and mouth ulcers. For example some participants indicated they had never had pain from their wisdom teeth but also that they had received treatment, such as gum cleaning, to wisdom teeth when they were causing pain. The importance of these contradictions will differ depending on the research question, to allow researchers the option of including or excluding these responses, the responses were all included in the dataset and new variables were generated to identify those participants with logical contradictions **(YPC2113 – YPC2120)**.

### Ethics policies

Ethical approval for the study was obtained from the ALSPAC Ethics and Law Committee and the Local Research Ethics Committees.

## Data availability

ALSPAC data access is through a system of managed open access. The steps below highlight how to apply for access to the data included in this data note and all other ALSPAC data. The datasets presented in this data note are linked to ALSPAC project number B2415, please quote this project number during your application. The ALSPAC variable codes highlighted in the dataset descriptions can be used to specify required variables.

1. Please read the ALSPAC access policy (PDF, 627kB) which describes the process of accessing the data and samples in detail, and outlines the costs associated with doing so.
2. You may also find it useful to browse our fully searchable research proposals database, which lists all research projects that have been approved since April 2011.
3. Please submit your research proposal for consideration by the ALSPAC Executive Committee. You will receive a response within 10 working days to advise you whether your proposal has been approved.

If you have any questions about accessing data, please email alspac-data@bristol.ac.uk.

The ALSPAC data management plan describes in detail the policy regarding data sharing, which is through a system of managed open access.

## Consent

Written informed consent was obtained from the parents of participating children after receiving a full explanation of the study. Children were invited to give assent where appropriate. Study members have the right to withdraw their consent for elements of the study or from the study entirely at any time. Full details of the ALSPAC consent procedures are available of the study website.

## Supplementary material

**Supplementary File 1. Me @ 23 questionnaire. Section J: Teeth.**

Pdf version of the relevant questionnaire section.
