# Supplementary material for: Age 23 years + oral health questionnaire in Avon Longitudinal Study of Parents and Children
Source: Wellcome Open Res. 2018 Apr 30;3:34. Originally published 2018 Mar 26. [Version 2] doi: 10.12688/wellcomeopenres.14159.2 (PMC5941244; doi:10.12688/wellcomeopenres.14159.2)
Supplement: Supplementary file 1 [file wellcomeopenres-3-15869-s0000.tgz › 433dd9fd-e3b0-45e7-883a-830ab4ee02de.pdf]

Introduction

This questionnaire is for completion by the study young person.

In answering these questions you will be helping Children of the 90s researchers who have been involved in putting this questionnaire together. The data you provide will be available to researchers across the world and will help in answering important questions on human development, health and disease.

Please remember that your answers to all these questions are confidential and will be processed using a unique ID number. All your personal details will be removed by Children of the 90s staff and researchers will not be able to link your answers back to you. Your data will only be shared with qualified researchers for research that has been approved by Children of the 90s.

Some questions may seem very similar to each other. This is because the combination of answers gives a clearer picture than one single answer. There may be questions that seem a bit strange and are not applicable to you because they are concerned with specific feelings or problems. We would be very grateful if you would try to answer all the questions but we understand if there are questions that you either prefer not to answer or are unable to answer. Please just leave these questions blank. There are no right or wrong answers.

If you require assistance in completing this questionnaire, please contact us via the details enclosed and we will be happy to make the necessary arrangements.

If you do not wish to complete this questionnaire, please leave it blank and return it to us in the prepaid envelope provided. We will then know not to send you any more reminders.

Thank you for taking the time to complete this questionnaire. To say thanks for taking part, we'll send you a £10 shopping voucher which you can spend online or on the high street. Whether you return your questionnaire complete or incomplete, we will also enter you into a prize draw to win one of three iPad Air 2 tablets.

To be entered into the prize draw we must have received your questionnaire by 5pm on Tuesday 3rd May 2016. We will contact winners within two weeks using the contact details we have on our database. Prize winners will receive their prizes up to six weeks after the draw has been held.

Filling in the Questionnaire

Please use a **black** pen. To answer questions simply put a cross in the circle/box which is most accurate in your opinion, like this:

⊗

☒

If you make a mistake, shade the circle/box in like this:

●

■

then cross the correct circle/box.

If you are answering questions which ask you to give further details, please make sure you write inside the boxes. If possible, please use CAPITAL LETTERS.

When writing numbers inside boxes, please don't touch the sides of the box. | 2 | 7 |

If you make a mistake when writing numbers inside boxes, please cross through the box and write your answer next to the box. ~~| 2 | 7 |~~ 2 8

If you do not want to answer a question, or if it does not apply to you, leave it blank.

There are no right or wrong answers.

There is a blank space available at the back of the questionnaire if you need additional space. If you use this sheet, please clearly indicate the question number you are answering.

Contents

		Page
Section A:	Social Media	4
Section B:	Sexual Experience	6
Section C:	Wellbeing	13
Section D:	Eating Behaviour	21
Section E:	Being a Parent	24
Section F:	Brothers and Sisters	25
Section G:	Smoking and E-cigarette Use	28
Section H:	Mental Health	35
Section I:	Traumatic Experiences	37
Section J:	Teeth	44
Section K:	Life Events	48
Section L:	Education and Employment	50
Section M:	Completing the Questionnaire	53

44394

Section J: Teeth

We are interested in the health of your teeth and mouth. We have asked some questions about your teeth before and it is useful to ask them again to see how things change over time.

Visiting your dentist and tooth decay

J1) When was the **last time** you went to the dentist?

- In the past year 1 O
- Between 1 and 2 years ago 2 O
- More than 2 years ago 3 O
- Never been 0 O
- Don't know 9 O

J2) What is the reason you **usually** go to the dentist?

- Regular routine check-ups (up to every 2 years) 1 O
- Occasional check up (less than every 2 years) 2 O
- Only when I have trouble with my teeth 3 O
- I never go to the dentist 0 O
- Don't know 9 O
- Another reason 4 O

J3) Were any of your teeth taken out for braces/traintracks/orthodontics?

Yes 1 O No 0 O

44394

J4) Here is a map of your mouth. Please look at your own mouth carefully with a mirror. For the moment ignore your wisdom teeth (shaded) if you have them. You may leave boxes blank if unsure.

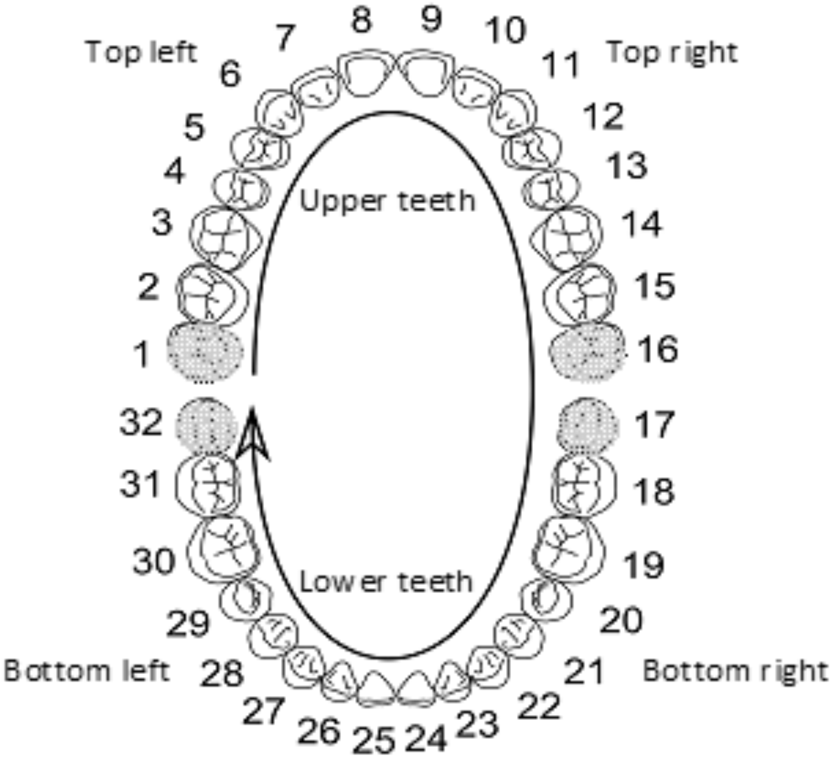

Universal numbering system (teeth diagram and numbers) by Kaligula and licensed under CC-BY-SA 3.0

a. Cross the boxes next to all teeth that have had fillings or other treatments like a cap or crown. Please note that a filling can be silver or white.

Top Left:	2 ☐	3 ☐	4 ☐	5 ☐	6 ☐	7 ☐	8 ☐
Top Right:	9 ☐	10 ☐	11 ☐	12 ☐	13 ☐	14 ☐	15 ☐
Bottom Right:	18 ☐	19 ☐	20 ☐	21 ☐	22 ☐	23 ☐	24 ☐
Bottom Left:	25 ☐	26 ☐	27 ☐	28 ☐	29 ☐	30 ☐	31 ☐

b. Cross the boxes next to all teeth that have been taken out.

Top Left:	2 ☐	3 ☐	4 ☐	5 ☐	6 ☐	7 ☐	8 ☐
Top Right:	9 ☐	10 ☐	11 ☐	12 ☐	13 ☐	14 ☐	15 ☐
Bottom Right:	18 ☐	19 ☐	20 ☐	21 ☐	22 ☐	23 ☐	24 ☐
Bottom Left:	25 ☐	26 ☐	27 ☐	28 ☐	29 ☐	30 ☐	31 ☐

44394

Third molars (wisdom teeth)

J5) Your wisdom teeth are at the very back of your mouth. Not everyone has them but, if you do, you will have the teeth shaded in the diagram at the back on each side (numbers 1, 16, 17 and 32). If you're unsure about these questions, please leave them blank.

a. Cross the boxes next to all wisdom teeth that **haven't** come through.

1 (top left) ☐ 16 (top right) ☐ 17 (bottom right) ☐ 32 (bottom left) ☐

b. Cross the boxes next to all wisdom teeth which **have** come through and have **not** caused you problems.

1 (top left) ☐ 16 (top right) ☐ 17 (bottom right) ☐ 32 (bottom left) ☐

c. Cross the boxes next to all wisdom teeth which **have** come through and **have** caused you problems or pain, even if these teeth have now been removed.

1 (top left) ☐ 16 (top right) ☐ 17 (bottom right) ☐ 32 (bottom left) ☐

J6) How many times:	Never	1	2-3	3-4	5 or more times	Don't know
a. Have you had pain from your wisdom teeth?	0 ○	1 ○	2 ○	3 ○	4 ○	9 ○
b. Have you had a course of antibiotics for problems with your wisdom teeth?	0 ○	1 ○	2 ○	3 ○	4 ○	9 ○
c. Have you had facial swelling from your wisdom teeth?	0 ○	1 ○	2 ○	3 ○	4 ○	9 ○

J7) Have you ever had to stay in a hospital bed, either during the day or overnight, because of problems from your wisdom teeth?

Yes 1 ○ No 0 ○ Don't know 9 ○

J8) Have you had any wisdom teeth removed?

Yes 1 ○ No 0 ○ Don't know 9 ○

J9) Have you had any other treatment to your wisdom teeth when they were causing pain, like cleaning around the gum or removing part of the gum?

Yes 1 ○ No 0 ○ Don't know 9 ○

44394

Mouth Ulcers

J10) Have you **ever** had mouth ulcers?

- Yes, but only once or twice 1 ○
- Yes, on several occasions 2 ○
- No 0 ○ ➡ **If <u>no</u>, go to section K**
- Don't know 9 ○

a. What age were you when you **first** noticed that you had mouth ulcers?

- Before I was a teenager (or under 12 years) 1 ○
- While I was a teenager (13-19) 2 ○
- In my 20s 3 ○
- Don't remember 9 ○

b. How often do you get mouth ulcers?

- Every month 1 ○
- Every 2-3 months 2 ○
- At least once every 6 months 3 ○
- At least once a year 4 ○
- Less than yearly 5 ○
- Don't remember 9 ○

44394

Section M: Completing the Questionnaire

M1) What is your date of birth? DD [ ][ ] / MM [ ][ ] / YYYY [1][9][9][ ]

M2) What is today's date? DD [ ][ ] / MM [ ][ ] / YYYY [2][0][1][ ]

Extra space for answering questions

Please clearly indicate the question number(s) your answer applies to.

44394

Me at 23+

Version 1   17/11/2015

Questionnaire Number

If you'd like to add a comment, please do so in the box below.
Please sign under your comment if you would like a response.

When completed, please send this back in the freepost envelope provided or post to:

Freepost (RRXX-UUZG-HTLK)
Children of the 90s
Oakfield House
15-23 Oakfield Grove
Bristol
BS8 2BN

Children of the 90s will send your thank you voucher within 4 weeks of receiving this questionnaire. Vouchers will be sent on our behalf by One4all Gift Cards. If you **do not** wish to receive your thank you voucher, please cross this box.

No Voucher ☐

If you **don't** wish to be entered into the prize draw, please cross this box.

No Prize Draw ☐

44394
