# Supplementary material for: Age 23 years + oral health questionnaire in Avon Longitudinal Study of Parents and Children
Source: Wellcome Open Res. 2018 Apr 30;3:34. Originally published 2018 Mar 26. [Version 2] doi: 10.12688/wellcomeopenres.14159.2 (PMC5941244; doi:10.12688/wellcomeopenres.14159.2)
Supplement: Peer review history

# Peer review: Referee response for version 2

**DOI:** 10.21956/wellcomeopenres.15869.r33009

**Published:** 2018-05-04

**Authors:** W. Murray Thomson

**Affiliations:** 1 School of Dentistry, University of Otago, Dunedin, New Zealand

The AU have done a nice job. My concern with the sentence about data collection was that it actually refers to data collection being collected - which remains awkward. Once that is fixed up (we collect data; we don't "collect data collection"...), the paper will be ready. I do not need to see this again.

I have read this submission. I believe that I have an appropriate level of expertise to confirm that it is of an acceptable scientific standard.

# Peer review: Referee response for version 1

**DOI:** 10.21956/wellcomeopenres.15404.r32906

**Published:** 2018-04-19

**Authors:** W. Murray Thomson

**Affiliations:** 1 School of Dentistry, University of Otago, Dunedin, New Zealand

*Overall comments*

 This is a useful data note. The actual writing could do with some close editing. The last sentence of the Abstract is particularly badly worded. The 4 research questions in the Introduction are also poorly worded, particularly the phrases “start to accurately predict” (maybe never?) and “start to predict”.

 Paras 2 and 3 of the Methods are on the same topic and should be combined. Para 7 – “data collection … were collected” is awkward.

 Re the Tables – “dont” should be “don’t”. And hyphenate “check up”.

*Is the rationale for creating the dataset(s) clearly described?*

 Yes

*Are the protocols appropriate and is the work technically sound?*

 Yes – insofar as the limitations of such limited self-report oral health data permit, of course. It would be useful to have some indication of the validity of the self-report tooth presence data – the AU could obtain such data with another, smaller sample which was then clinically examined by calibrated dental examiners. People will find it more straightforward to publish the data from the parent study if such information is available, so it would be a sound investment of time and money.

*Are sufficient details of methods and materials provided to allow replication by others?*

 Yes.

*Are the datasets clearly presented in a useable and accessible format?*

 Mostly. It would be useful to have a Table summarising the missing data; that is, a Table listing each variable and the number of valid and missing responses so that the reader could see it at a glance.

 The % in Tables 4 and 5 should be presented to only 1 decimal place (as in the other Tables). Using 2 implies a degree of accuracy which is inappropriate here. In Tables 4 and 5, FDI tooth notation should be used, so that “UR1” becomes tooth 11, etc. The column headings “Right and “Left” should be centred.

I have read this submission. I believe that I have an appropriate level of expertise to confirm that it is of an acceptable scientific standard, however I have significant reservations, as outlined above.

# Response

**Published:** 2018-04-26

**Authors:** Tom Dudding

**Affiliations:** University of Bristol, UK

We thank the referee for their helpful comments and provide a point-by-point response.

1. The actual writing could do with some close editing. The last sentence of the Abstract is particularly badly worded. The 4 research questions in the Introduction are also poorly worded, particularly the phrases “start to accurately predict” (maybe never?) and “start to predict”.

**Reply: **We have edited the manuscript to amend those areas where there is poor wording. The final line of the abstract now reads **“This round of data collection follows on from similar questionnaires at ages 7, 10 and 17 years. The ALSPAC study provides an opportunity to combine this oral health data with extensive prospective phenotype, genetic, epigenetic and metabolomic data from the participants, their mothers and fathers.” **The first and third research question have been re-phrased to **“Can oral health in adolescence predict poor health outcomes in later life and if so at what stage is this detectable?” **and **“At what stage do oral hygiene behaviours and beliefs predict periodontal outcomes? Are patterns of behaviour in childhood or early adult life more important?” **respectively.

1. Paras 2 and 3 of the Methods are on the same topic and should be combined. Para 7 – “data collection … were collected” is awkward.

**Reply: **We have combined paragraphs 2 and 3 of the Methods and reworded paragraph 7 to read **“..data collection for the online questionnaires was collected and managed using REDCap electronic data capture tools…”**

1. Re the Tables – “dont” should be “don’t”. And hyphenate “check up”.

**Reply: **In all tables “dont” has been replaced by **“don’t”** and “check up” is now hyphenated in table 2.

1. *Are the protocols appropriate and is the work technically sound?*
  Yes – insofar as the limitations of such limited self-report oral health data permit, of course. It would be useful to have some indication of the validity of the self-report tooth presence data – the AU could obtain such data with another, smaller sample which was then clinically examined by calibrated dental examiners. People will find it more straightforward to publish the data from the parent study if such information is available, so it would be a sound investment of time and money.

**Reply: **We thank the referee for this comment and agree that this dataset would be improved by validating it against a clinical sample. Although this is not possible for this round of data collection the authors would like to carry out a clinical validation of the next round of oral health questionnaires in the ALSPAC study.

1. *Are the datasets clearly presented in a useable and accessible format?*

Mostly. It would be useful to have a Table summarising the missing data; that is, a Table listing each variable and the number of valid and missing responses so that the reader could see it at a glance.

**Reply: **We have added a table (Table 14) to the revised data note that provides the number and percentage of valid and missing responses to each question other than those summarised in Table 4 (reported frequency of fillings and extractions) and Table 5 (wisdom teeth). These variables have not been included in Table 14 as no information additional to Tables 4 and 5 would be presented.

1. The % in Tables 4 and 5 should be presented to only 1 decimal place (as in the other Tables). Using 2 implies a degree of accuracy which is inappropriate here. In Tables 4 and 5, FDI tooth notation should be used, so that “UR1” becomes tooth 11, etc. The column headings “Right and “Left” should be centred.

**Reply: **We have altered tables 4 and 5 to present the data to 1 decimal place. Thank you for your comment regarding using the FDI notation, we feel this is a valuable addition to the table. We have decided to include it in addition to, as opposed to instead of, the letters and numbers notation, as we feel this latter notation is more useful to non-dentally trained users. The column headers “Left” and “Right” have been centred.

# Peer review: Referee response for version 1

**DOI:** 10.21956/wellcomeopenres.15404.r32474

**Published:** 2018-04-19

**Authors:** Thomas Dietrich

**Affiliations:** 1 School of Dentistry, Institute of Clinical Sciences, University of Birmingham, Birmingham, UK

This manuscript describes an oral health related follow-up questionnaire collecting self-reported  data on dental attendance, tooth restorations and extractions, third molars and mouth ulcers up to 4,222 from participants in the Avon Longitudinal Study of Parents and Children (ALSPAC), who are now approximately 23 years of age. The rationale, items and basic response frequencies are clearly describes and so is the accessibility of this resource. The authors may wish to consider to add a flow-chart describing how they arrived at the final sample size for this round of follow-up, as the description in the text is at times hard to follow.

I have read this submission. I believe that I have an appropriate level of expertise to confirm that it is of an acceptable scientific standard.

# Response

**Published:** 2018-04-26

**Authors:** Tom Dudding

**Affiliations:** University of Bristol, UK

We thank the referee for their comments and the suggestion of the addition of a flow-chart to clearly show the final sample size. This has now been added as an additional figure (Figure 1).
